# Multiple Venous Thromboembolism Pharmacologic Agents Are Associated with an Increased Risk for Early Postoperative Complications following a Total Joint Arthroplasty

**DOI:** 10.1155/2022/8318595

**Published:** 2022-02-07

**Authors:** Jonathan H. Shaw, Luke D. Wesemann, Omar M. Kadri, Clifford M. Les, Wayne T. North, Michael A. Charters

**Affiliations:** ^1^Department of Orthopaedic Surgery, Henry Ford Hospital, 2799 West Grand Boulevard, Detroit, MI 48202, USA; ^2^Wayne State University School of Medicine, 540 E Canfield St, Detroit, MI 48201, USA

## Abstract

The purpose of this study was to determine the effect that concurrent venous thromboembolism (VTE) medications had on early outcomes following primary total joint arthroplasty (TJA). 2653 total knee and hip arthroplasties were reviewed at a tertiary medical center. The study performed a multivariable comparison of outcomes in patients on 2 or more VTE medications, as well as a logistic regression on outcomes following each addition of a VTE medication postoperatively (number of VTE medications was 1–4). Controlling for gender, age, body mass index, and preoperative American Society of Anesthesiologists score throughout the analysis, patients who received 2 or more VTE prophylaxis medications had increased LOS (*p* < 0.001), transfusions (*p* < 0.001), emergency department visits (*p*=0.001), readmissions (*p* < 0.001), 90dPOE (*p* < 0.001), and PE (*p* < 0.001). Every additional postoperative VTE medication incrementally increased the risk for longer LOS (*p* < 0.001), transfusions (*p* < 0.001), 90dPOE (*p* < 0.001), deep vein thrombosis (*p*=0.049), PE (*p* < 0.001), emergency department visits (*p*=0.005), and readmission (*p*=0.010). Patients on multiple VTE medications following TJA demonstrate significantly poorer outcomes. The current study's findings caution the use of multiple VTE medications whenever possible immediately following a TJA.

## 1. Introduction (DVT: Deep Vein Thrombosis; PE: Pulmonary Embolism; THA: Total Hip Arthroplasty; TJA: Total Joint Arthroplasty; TKA: Total Knee Arthroplasty; VTE: Venous Thromboembolism)

Since 2013, the number of total joint arthroplasties (TJAs) performed in the United States exceeds 1,000,000 annually. Additionally, from 2000 to 2014, the annual volume of total hip arthroplasties (THAs) increased 132%, and total knee arthroplasties (TKAs) increased 148% [1]. THA and TKA are successful surgical procedures in terms of cost-effectiveness and the impact on the quality of life; however, complications such as venous thromboembolism (VTE) can occur perioperatively leading to adverse outcomes [2, 3]. As a result, postoperative VTE prophylaxis has become routine following TJA. Without VTE prophylaxis, both THA and TKA have a deep vein thrombosis (DVT) or pulmonary embolism (PE) occurrence rate of up to 85% [4]. There is consensus regarding the need for VTE prophylaxis, but the method of anticoagulation differs among total joint surgeons. The 2017 American Academy of Orthopedic Surgeons guidelines for VTE prophylaxis in patients undergoing TKA and THA recommend a wide array of prophylactic agents, yet there is no evidence for distinct superiority of a certain agent [5, 6]. The survey determined that low-molecular-weight heparin, warfarin, aspirin, and fondaparinux were the most commonly used prophylaxis agents.

Complications after THA and TKA are multifaceted issues often with significant consequences to the patient's health, including an increased morbidity and mortality. Much attention has recently been drawn to these complications as payment structures are evolving towards bundle payments. In addition to different preferences in postoperative VTE prophylaxis, patients who present for a TJA may have several VTE medications on their medication administration record secondary to comorbidities such as cardiovascular or cerebrovascular disease. There are currently limited data on the management of postoperative VTE prophylaxis for patients undergoing TJA who present with multiple preoperative VTE agents. To date, no study has compared postoperative outcomes for patients on multiple VTE medications postoperatively following a total joint arthroplasty.

The purpose of this study was to determine the effect concurrent VTE medications had on early outcomes following primary TJA. It is hypothesized that each additional VTE medication postoperatively increases the patient's risk of bleeding events, resulting in longer hospital stays and increased rates of postoperative complications.

## 2. Materials and Methods

We performed a retrospective cohort analysis of 2653 patients that underwent TKA and THA by multiple surgeons at a university affiliated, healthcare system in Southeast Michigan from January 2014 to December 2016. After institutional review board approval, data were standardized and collected by the study institution for inclusion in the Michigan Arthroplasty Registry Collaborative Quality Initiative.

Patient comorbidities were controlled for using the American Society of Anesthesiologists (ASA) score. Patient demographics collected in the chart review included age, sex, body mass index (BMI), preoperative ASA, laterality, and surgery performed (THA/TKA). Postoperative outcomes included length of stay (LOS), emergency department visit with no readmission, readmission (<30 days postoperatively), 90-day postoperative events, intraoperative events, and postoperative blood transfusion. Postoperative events included DVT, PE, hematoma, joint space infection, and death. The VTE prophylaxis agents recorded were aspirin, unfractionated or low-molecular-weight heparin, direct thrombin or factor Xa inhibitor, other antiplatelets, and warfarin.

The inclusion criteria consisted of primary joint replacement (THA or TKA). The study excluded partial joint arthroplasty, arthroplasty due to a traumatic event, revision arthroplasty, and patients who underwent multiple joint replacements within the selected period.

Postoperatively, patients received 24 hours of antibiotic prophylaxis and were mobilized with physical therapy on postoperative day 0-1. Patients received tranexamic acid per hospital protocol. The protocol consists of 1 g of intravenous tranexamic acid before incision in hips or closure of the incision. If patients had contraindication to intravenous tranexamic acid, then 2 g of topical tranexamic acid was placed into the incision at the time of closure. During the time period of the study, patients were transfused when hemoglobin was less than 8 mg/dL if symptomatic or if hemoglobin was less than 7 mg/dL. Each patient's postoperative anticoagulation followed the hospital protocol.

Throughout the study period following surgery, all patients received at least one VTE prophylaxis medication, a sequential compression device, and early mobilization, per institutional protocol. Patients at the tertiary medical center with no history of prior DVT or PE were given aspirin 81 mg twice a day for 4 weeks starting postoperative day 0. If the patient could not take aspirin, then they received enoxaparin 40 mg daily for 3 weeks. High-risk patients were given enoxaparin 30 mg twice a day for 4 weeks. If the patient was on warfarin at home, then enoxaparin 40 mg daily was administered on postoperative day 1, and warfarin was restarted on postoperative day 0. Enoxaparin was discontinued once international normalized ratio was at a therapeutic level (2.5). Patients on a factor Xa inhibitor were given half the dose on postoperative day 1 and transitioned to a full dose on postoperative day 7.

Patients were seen by either their primary care physician, an optimization clinic, or a specialist (i.e., cardiology) preoperatively before undergoing TJA. At this appointment, basic preoperative labs including complete blood count, basic metabolic profile, and coagulation profile were part of determining a patient's health status. A patient was deemed acceptable for elective surgery with hemoglobin above 10 gm/dL with a surgical preference of 12 gm/dL. A patient's coagulopathy was corrected preoperatively based on an INR level less than 2. Finally, a patient was considered optimal with a platelet count of greater than 100,000 platelets per microliter, and if below, it had to be cleared with a hematologist.

### 2.1. Statistical Analysis

All statistical tests were run on SigmaStat 12.3 (Systat Software Inc., San Jose, CA). Alpha in all cases was set to 0.05. Continuous variables (BMI, age, and preoperative ASA score) were examined as parametric *T*-tests and as nonparametric rank-sum tests. Categorical variables (case joint, side, race, gender, and preoperative ASA score) were examined as chi-square analyses.

Risk factors, considered as binary output variables (LOS, considered as above or below the median value for this cohort, transfusion, emergency department visit, 30-day readmission, urinary tract infection, DVT, PE, hematoma, joint space infection, other reason for returning to the operating room, or death), were examined as multiple logistic regressions, taking into account BMI, age, gender, preoperative ASA score, and number of VTE medications. A similar analysis was performed, instead examining the effect of 1 versus 2 or more postoperative VTE medications. Finally, the analysis was performed again, examining the effect of the total number of postoperative VTE medications used (considered as a continuous variable). For this analysis, LOS (days) was also considered as a continuous variable in a multiple linear regression.

## 3. Results

A total of 2653 participants underwent primary THA or TKA during the study period. The study group consisted of 1666 TKAs (62.8%) and 987 THAs (37.2%). Eight hundred eighty-one patients received multiple VTE prophylaxis medications postoperatively (33.2%; 537 TKAs and 344 THAs), while 1772 received only 1 modality (66.8%; 1129 TKAs and 643 THAs) (Table 1).

Demographic data (BMI, case joint, side, race, gender, age, and preoperative ASA score) were compared between patients who received 2 or more inpatient prophylaxis (Table 2). Patients on 2 or more VTE medications postoperatively were more likely to be older (*p* < 0.001) and have a higher preoperative ASA score (*p* < 0.001).

After controlling for gender, age, BMI, and preoperative ASA score, patients who received 2 or more medications for VTE prophylaxis had an increased LOS (OR: 2.305, *p* < 0.001) as well as significant risk for 90-day postoperative events (OR: 0.595, *p* < 0.001) and PE (OR: 9.225, *p* < 0.001) (Table 3). These patients were also twice as likely to be transfused (1.993, *p* < 0.001) and had a higher occurrence of emergency department visits without readmission (OR: 1.546, *p*=0.001) and readmissions (OR: 1.799, *p* < 0.001). Interestingly, there were 144 patients of the 881 patients receiving multiple VTE prophylaxis medications who received 3 or 4 different modalities. Of those patients, 131 (91.0%) of them were recorded to have 3 or more VTE prophylaxis medications.

Continuing to control for BMI, age, gender, and preoperative ASA score, for every additional postoperative VTE medication, ranging 1 to 4, the risk for longer LOS (2.039, *p* < 0.001), chances for inpatient transfusion (1.646, *p* < 0.001), 90-day postoperative events (1.325, *p* < 0.001), DVT (1.541, *p*=0.049), PE (8.147, *p* < 0.001), emergency department visit (1.329, *p*=0.005), and readmission (1.379, *p*=0.010) incrementally increased (Table 4).

## 4. Discussion

As the number of TJAs continues to increase, it is important to continue to stratify patients and reduce postoperative complications, especially as the emphasis for value-based care drives the healthcare system. This multivariate analysis demonstrated that patients on multiple VTE prophylaxis agents postoperatively were at increased risk for complications after TJA. In addition, patients receiving 2 or more VTE prophylaxis agents postoperatively demonstrated increased LOS as well as increased rates of postoperative transfusion, urinary tract infection, PE, emergency department visits without readmission, and readmission. The logistic regression analysis demonstrated that these risks increase for every additional prophylactic VTE medication.

These findings demonstrate a subset of patients that can potentially be optimized further prior to TJA in order to reduce the risk of increased morbidity and cost. Referencing a study published in the *Journal of Thrombosis and Hemostasis* seeking to objectively define major bleeding in surgical patients, this study did find an association with an increased rate of transfusion in patients on 2 or more anticoagulants postoperatively [7]. Hallevi and collaborators compared different regimens for starting long-term anticoagulation after cardioembolic stroke and found that enoxaparin or heparin bridging increased bleeding risks in cardioembolic stroke patients [8]. Similar to this study, Leijtens et al. found an increased risk of transfusion, hematoma, readmission, LOS, and periprosthetic joint infection in their patients requiring perioperative heparin bridging [9]. In TJA patients requiring blood transfusion, one study found a significant association between blood transfusion and risk of periprosthetic joint infection [10]. Ferraris et al. presented the risks that come with intraoperative and postoperative transfusion. Not only are patients exposed to increased risks of allergic reactions, infectious diseases, lung injury, and hemolytic reactions but also patients who received a transfusion postoperatively had adverse health outcomes, such as increased mortality [11]. With any TJA, the risk of VTE must be balanced with the risk of bleeding complications [12–18]. Patients should be counseled on these risks, and further research is required to continue to decrease this risk. This study demonstrated that transfusion rates were increased with every additional VTE medication that patients took postoperatively.

The emphasis on value-based healthcare continues to increase. This study demonstrated several postoperative events including increased length of stay, postoperative ED visits, and readmission rates that have been heavily scrutinized in the arthroplasty literature due to the higher costs on the healthcare. Surgical complications cause readmission to cost an average of $45,901 for TKA and $31,880 for THA [19]. Due to the current reimbursement policy, hospitals receive an average reimbursement of $9,423 from Medicare/Medicaid for TKA and $20,517 for THA [19]. Concomitantly, Chen et al. investigated costs of transfusion postoperatively after total knee and hip arthroplasties [16]. They demonstrated that patients who received blood transfusions after TKA ($61,548.25) were higher than those that did not receive blood transfusions ($51,768.58), and hips were $82,588.60 versus $64,845.60. They correlated this increase cost with an increased LOS, which is similar to the findings of this study. Moreover, postoperative ED visits with or without readmission greatly increase the cost of TJA [20, 21]. Unfortunately, many of the current bundling models are limited in the risk-adjustment techniques for factors such as increased ASA score, preoperative therapy, and subsequent major complications. Thus, the onus falls on the surgical team to optimize patients prior to TJA in order to decrease the cost associated with the surgical episode. As the healthcare landscape continues to change, mitigation of these risks will be of utmost importance. Further studies will be needed in order to evaluate the cost savings associated with patient optimization prior to TJA.

There are several limitations to the present study. Since there is a low occurrence of the complications studied, a cohort size of 2653 provides adequate power to find a difference. Yet since, the lowest possible population expected to produce a clinical difference was not calculated, one may dispute significant differences as this cohort size may not be strong enough. A retrospective study always has its limitations. The collected information cannot be verified for accuracy, which is based on the health professional who recorded the information. Along with the collection of large databases, the dose and frequency of the medications cannot be verified for more accurate conclusions between different strengths and dose effects. Finally, discrete laboratory values preoperatively were not collected, and this is certainly a limitation of this retrospective review. This limitation does not allow the authors to definitely conclude the incidence of patients undergoing TJA with laboratory values that did not meet the health system protocol stated in the methods section. A strength of this study is that this study received no industry funding or support.

## 5. Conclusions

This study identified the association of administering multiple antithrombotic agents postoperatively with an increased rate of poor postoperative outcomes following TJA. The results demonstrate the fine balance between the need for VTE prophylaxis and the risk for other postoperative complications. It reinforces this balance by demonstrating that patients on multiple VTE medications postoperatively have a significantly higher risk for a PE and DVT, and patients on 2 or more VTE modalities have an increased risk of transfusion. Logistic regression suggests that if patients receiving 2 or more VTE modalities decreased 1 pharmacologic agent, their risk of a transfusion, emergency department visit without readmission, and readmission would significantly decrease. All of the findings demonstrate that additional VTE prophylaxis postoperatively has a significant effect in outcomes following TKA and THA.

## Figures and Tables

**Table 1:** Postoperative anticoagulants prescribed.

Number of postoperative VTE medications	Aspirin	Unfractionated heparin or LMWH	Direct factor Xa or II inhibitor	Antiplatelet excluding ASA	Warfarin	Total patients

1	995 (56.2%)	119 (6.7%)	519 (29.3%)	0	158 (8.9%)	1772
2	539 (73.1%)	737 (100%)	371 (50.3%)	0	280 (38.0%)	737
3	125 (90.6%)	134 (97.1%)	26 (18.8%)	1 (0.7%)	128 (92.8%)	138
4	6 (100%)	6 (100%)	5 (83.3%)	1 (16.7%)	6 (100%)	6

**Table 2:** Demographics of patients on 2 or more VTE medications postoperatively.

Parameter	Test	*p* value	2 or more inpatient prophylaxis (*N* = 881)	Others (*N* = 1779)

Body mass index	*T*-test	0.122	31.6 (6.5)	32.1 (6.7)
	Rank sum	0.100	30.8 (27.1–35.5)	31.2 (27.4–36.4)

Case joint	Chi-square	0.165	TKA = 537	TKA = 1135
			THA = 344	THA = 644

Side	Chi-square	0.987	Left = 408	Left = 821
			Right = 455	Right = 923
			Both = 18	Both = 35

Race	Chi-square	0.996	Caucasian = 732	Caucasian = 1476
			AA = 111	AA = 226
			Unknown = 38	Unknown = 76

Gender	Chi-square	0.143	Male = 374	Male = 701
			Female = 507	Female = 1078

Age	*T*-test	<0.001	68.6 (10.1)	64.7 (10.4)
	Rank sum	<0.001	69.0 (62.0–76.0)	65.0 (58.0–72.0)

Preoperative ASA (categorical)	Chi-square	<0.001	1 = 3	1 = 17
			2 = 156	2 = 489
			3 = 625	3 = 1165
			4 = 96	4 = 107

Preoperative ASA (continuous)	*T*-test	<0.001	2.9 (0.5)	2.8 (0.6)
	Rank sum	<0.001	3.0 (3.0–3.0)	3.0 (2.0–3.0)

AA: African American; ASA: American Society of Anesthesiologists; THA: total hip arthroplasty; TKA: total knee arthroplasty; VTE: venous thromboembolism. Patients on 2 or more VTE medications postoperatively were more likely to be older and have a higher preoperative ASA score.

**Table 3:** Multivariate analysis of 2 or more venous thromboembolism medications postoperatively.

Factors	Lower	Upper	Odds	*p* value

Length of stay (logistic regression)	1.855	2.863	2.305	<0.001
Transfusion	1.439	2.761	1.993	<0.001
Emergency department visit	1.186	2.015	1.546	<0.001
30-day readmission	1.288	2.511	1.799	<0.001
90-day postoperative events	1.268	1.516	1.405	0.001
Urinary tract infection	1.063	8.379	2.984	0.038
Deep venous thrombosis	0.559	1.976	1.051	0.878
Pulmonary embolism	2.586	32.901	9.225	<0.001
Hematoma	0.802	3.793	1.744	0.161
Joint space infection	0.785	4.752	1.931	0.152
Other return to the operating room	0.676	1.772	1.095	0.712
Death	0.25	13.4	1.829	0.552

Controlling for gender, age, joint, BMI, and preoperative ASA, patients on multiple anticoagulants in the hospital were at an increased risk for length of stay, pulmonary embolism, no 90-day postoperative events, readmission, and transfusion.

**Table 4:** Logistic regression analysis on increasing the number of VTE medications postoperatively.

Event	Risk factors				
	BMI	Gender (female)	Age	ASA score	Inpatient postoperative prophylaxis count

Length of stay (logistic regression)	0.002 (1.021, 1.008–1.035)	<0.001 (2.208, 1.861–2.620	<0.001 (1.042, 1.033–1.051)	<0.001 (1.680, 1.435–1.966)	<0.001 (2.039, 1.747–2.378)
No 90-day postoperative events	0.719	0.315	0.876	0.014 (0.789 0.653–0.954)	<0.001 (0.675, 0.578–0.789)
Emergency department visit	0.629	0.772	0.525	0.223	0.005 (1.329, 1.088–1.623)
Readmit	0.465	0.416	0.010 (1.023, 1.006–1.041)	0.002 (1.668, 1.215–2.292)	0.010 (1.379, 10.78–1.762)
DVT	0.120	0.504	0.184	0.774	0.049 (1.541, 1.002–2.371)
UTI	0.962	0.448	0.267	0.444	0.011 (2.306, 1.212–4.389)
Death	0.954	0.309	0.598	0.003 (33.409, 3.318–336.402)	0.897
PE	0.183	0.761	0.706	0.186	<0.001 (8.147, 4.205–15.785)
Other return to the operating room	0.525	0.028 (1.713, 1.059–2.773)	0.003 (0.967, 0.946–0.989)	0.786	0.331
Hematoma	0.155	0.669	0.685	0.333	0.282
JSI	0.225	0.911	0.122	0.371	0.543
Transfusion	<0.001 (0.943, 0.918–0.969)	0.163	0.252	0.002 (1.611, 1.184–2.193)	<0.001 (1.646, 1.308–2.072)

Single values represent odds ratios. Multiple values represent *p* value (odds ratio, confidence interval). ASA: American Society of Anesthesiologists; BMI: body mass index; DVT: deep vein thrombosis; JSI: joint space infection; PE: pulmonary embolism; UTI: urinary tract infection; VTE: venous thromboembolism. Controlling for BMI, gender, age, and preoperative ASA score, additional postoperative VTE medication, ranging 1 to 4, incrementally increases the risk for longer length of stay, chances for inpatient transfusion, 90-day postoperative event, DVT, PE, emergency department visit, and readmission.

## Data Availability

The data that support the findings of this study are available from the corresponding author, LW, upon reasonable request.
